# Safety, Tolerability, Pharmacokinetics, and Pharmacodynamics of Coadministered Ruxolitinib and Artemether-Lumefantrine in Healthy Adults

**DOI:** 10.1128/AAC.01584-21

**Published:** 2022-01-18

**Authors:** M. Farouk Chughlay, Karen I. Barnes, Myriam El Gaaloul, Nada Abla, Jörg J. Möhrle, Paul Griffin, Paul van Giersbergen, Stephanie E. Reuter, Hayley B. Schultz, Anita Kress, Peter Tapley, Rebecca A. Webster, Timothy Wells, James S. McCarthy, Bridget E. Barber, Louise Marquart, Michelle J. Boyle, Christian R. Engwerda, Stephan Chalon

**Affiliations:** a Medicines for Malaria Venture, Geneva, Switzerland; b Division of Clinical Pharmacology, Department of Medicine, University of Cape Town, Cape Town, South Africa; c QIMR Berghofer Medical Research Institute, Brisbane, Queensland, Australia; d Q-Pharm Pty, Ltd., Herston, Queensland, Australia; e University of Queensland, Brisbane, Queensland, Australia; f Mater Health Services, South Brisbane, Queensland, Australia; g Van Giersbergen Consulting, Wuenheim, France; h UniSA Clinical and Health Sciences, University of South Australia, Adelaide, South Australia, Australia; i Swiss BioQuant, Reinach, Switzerland; j TetraQ, Brisbane, Queensland, Australia

**Keywords:** artemether-lumefantrine, clinical trial, healthy volunteers, malaria, pharmacokinetics, phase 1 study, ruxolitinib, signal transducer and activator of transcription 3

## Abstract

Despite repeated malaria infection, individuals living in areas where malaria is endemic remain vulnerable to reinfection. The Janus kinase (JAK1/2) inhibitor ruxolitinib could potentially disrupt the parasite-induced dysfunctional immune response when administered with antimalarial therapy. This randomized, single-blind, placebo-controlled, single-center phase 1 trial investigated the safety, tolerability, and pharmacokinetic and pharmacodynamic profile of ruxolitinib and the approved antimalarial artemether-lumefantrine in combination. Ruxolitinib pharmacodynamics were assessed by inhibition of phosphorylation of signal transducer and activator of transcription 3 (pSTAT3). Eight healthy male and female participants ages 18 to 55 years were randomized to either ruxolitinib (20 mg) (*n = *6) or placebo (*n = *2) administered 2 h after artemether-lumefantrine (80/480 mg) twice daily for 3 days. Mild adverse events occurred in six participants (four ruxolitinib; two placebo). The combination of artemether-lumefantrine and ruxolitinib was well tolerated, with adverse events and pharmacokinetics consistent with the known profiles of both drugs. The incidence of adverse events and artemether, dihydroartemisinin (the major active metabolite of artemether), and lumefantrine exposure were not affected by ruxolitinib coadministration. Ruxolitinib coadministration resulted in a 3-fold-greater pSTAT3 inhibition compared to placebo (geometric mean ratio = 3.01 [90% confidence interval = 2.14 to 4.24]), with a direct and predictable relationship between ruxolitinib plasma concentrations and %pSTAT3 inhibition. This study supports the investigation of the combination of artemether-lumefantrine and ruxolitinib in healthy volunteers infected with Plasmodium falciparum malaria. (This study has been registered at ClinicalTrials.gov under registration no. NCT04456634.)

## INTRODUCTION

Malaria remains a major global health issue and a significant problem in tropical and subtropical regions of the world (1). A key impediment to malaria eradication is the poor understanding of host immunity against *Plasmodium* species. Antibodies with specific functional properties are required to mediate host immunity (2–10). However, in individuals living in areas where malaria is endemic, although antiparasitic responses are often present, they do not confer robust protective immunity (11–13). Evidence indicates the presence of parasite-induced immunoregulatory mechanisms that may protect tissue from acute inflammation, but also promote the development of atypical B cells, suboptimal function of CD4^+^ T follicular helper (Tfh) cells and Tbet^+^ CD4^+^ T (Th1) cells, and autologous interleukin-10 (IL-10) production by the latter CD4^+^ T cell subset (10, 14–19).

Type I interferons (IFNs) are important regulators of IL-10 production by Tr1 cells (20). Type I IFNs signal through the common IFN-α receptor (IFNAR), consisting of IFNAR1 and IFNAR2 chains. The IFNAR signals via signal transducers and activators of transcription 1 and 2 (STAT1 and STAT2) and has been shown to mediate diverse functions during a variety of infections (21–23).

A causal link between immune dysregulation and recurrent infection or severe malaria in individuals living in areas where malaria is endemic has not been investigated. However, single nucleotide polymorphisms (SNPs) in the *IFNAR1* gene (loci 17470, L168 V, and 272354) were associated with an increased risk of severe malaria in The Gambia, and subsequently a Chr21q22.11 C>G SNP (IFNAR1 272354c-g) at position −576 relative to the transcription start was strongly associated with susceptibility to severe malaria in Gambian, Kenyan, and Vietnamese case-control studies (24, 25). Also, in malaria naive individuals in volunteer infection studies (VIS), type I IFNs suppressed innate immune cell function and parasite-specific CD4^+^ T cell gamma IFN (IFN-γ) production, and promoted the development of parasite-specific Tr1 cells (20). Murine studies support a link between type I IFN and the pathogenesis of experimental cerebral malaria (26), suppression of CD4^+^ T cell-dependent parasite control (27, 28), and the expansion of IL-10-producing Th1 (Tr1) cells (29). Thus, type I IFNs are key immunomodulatory molecules for the development of antiparasitic immune responses to P. falciparum. Targeting this pathway is a possible strategy to overcome established or developing immunoregulatory networks to enhance immunity against malaria.

Ruxolitinib is an orally administered small molecule inhibitor of Janus-Associated Kinase 1 (JAK1) and JAK2 approved for the treatment of intermediate or high-risk myelofibrosis and polycythemia vera in adults and of steroid-refractory acute graft-versus-host disease in patients 12 years and older and has also been safely and effectively used in children with type I interferonopathy (30–33). The JAK family of tyrosine kinases are closely associated with cytokine receptors, such as the type I IFN receptor; JAK becomes phosphorylated after cytokines bind to these receptors that in turn phosphorylate STAT, mediating signal transduction to the cell nucleus (34). In monocytes and T cells, the JAK 1/2-mediated phosphorylation of STAT3 (pSTAT3) that occurs after binding of IL-6 to its receptor can be used to measure the pharmacodynamic effect of ruxolitinib (30, 35).

Ruxolitinib has been shown to block type I IFN signaling in a range of human diseases (30, 33), and the potential for ruxolitinib to disrupt the parasite-induced dysfunctional immune response in malaria requires investigation. For example, ruxolitinib could be coadministered with antimalarial therapy for a first malaria episode to potentially prevent the development of immune dysregulation and reduce the risk of recurrent infection or severe disease. However, ruxolitinib safety and efficacy has not been evaluated when coadministered with antimalarial medication.

In this study, we investigated the safety, tolerability, and pharmacokinetic and pharmacodynamic profile for the combination of ruxolitinib and the approved artemisinin-based combination, artemether-lumefantrine, widely used for the treatment of uncomplicated malaria. Ruxolitinib pharmacodynamic activity was assessed by measuring pSTAT3 inhibition (35). This study aimed to facilitate future research to evaluate the immune enhancing potential of ruxolitinib when given with the approved antimalarial artemether-lumefantrine for the treatment of uncomplicated P. falciparum malaria.

## RESULTS

### Participants.

**FIG 1:**
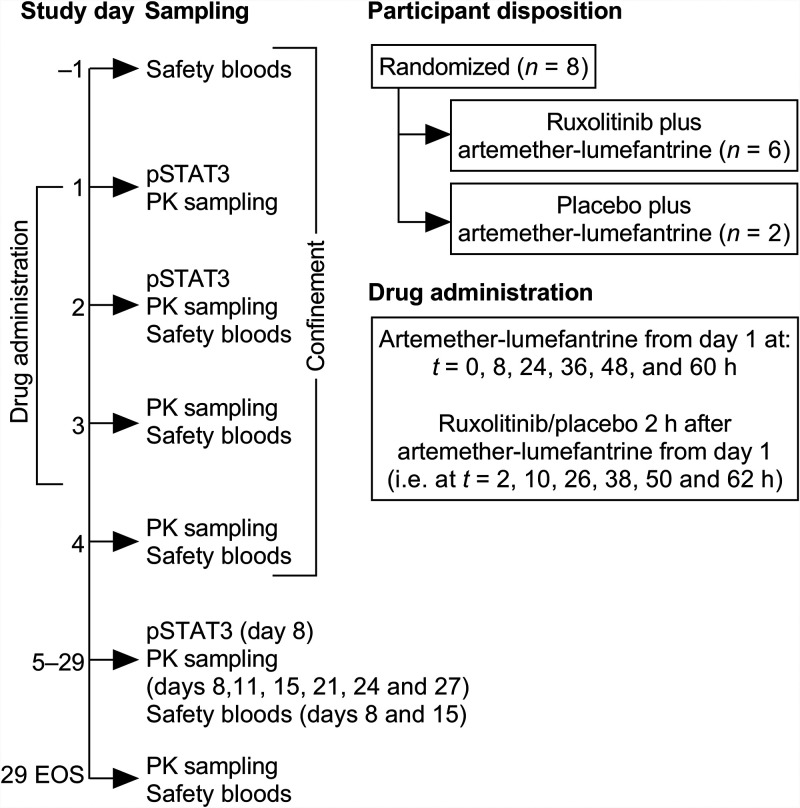
Study design and randomization. PK, pharmacokinetics; EOS, end of study.

Eight participants were randomized, six to artemether-lumefantrine plus ruxolitinib and two to artemether-lumefantrine plus placebo (Fig. 1 and Table 1). All participants had valid drug plasma concentration and pSTAT3 data and were included in the safety, pharmacokinetic, and pharmacodynamic analyses. One participant who received artemether-lumefantrine plus ruxolitinib withdrew consent on day 11; data for this participant were available up to day 8. Thus, seven participants completed the study.

**TABLE 1:** Demographic characteristics^*a*^

Characteristic	AL+RUX (*n *=* *6)	AL+placebo (*n *=* *2)
Mean age, yrs (SD)	26.3 (11.8)	30.0 (12.7)
Mean wt, kg (SD)	66.3 (16.0)	78.9 (6.6)
No. (%) self-declared ethnicity		
Caucasian	5 (83.3)	2 (100)
Aboriginal	1 (16.7)	0
No. of male/female participants	4/2	1/1

a AL, artemether-lumefantrine; RUX, ruxolitinib.

### Adverse events.

A total of six participants experienced adverse events (Table 2). All adverse events were of mild severity. There were no clinically important differences in the incidence or severity of adverse events between the two study groups (Table 2). In the artemether-lumefantrine plus ruxolitinib group, only one adverse event (headache) was considered drug related, whereas headache and maculopapular rash were considered drug related in the artemether-lumefantrine plus placebo group. The maculopapular rash in one participant appeared 12 days after first drug administration and resolved within 3 days with the application of topical corticosteroids. There were no adverse events that led to premature discontinuation, no deaths, and no other serious adverse events.

**TABLE 2:** Summary of all treatment-emergent adverse events of any cause

Adverse event	No. (%) of participants with adverse event in study group^*a*^	
	AL+RUX (*n *=* *6)	AL+placebo (*n *=* *2)
Any adverse event	4 (66.7)	2 (100)
Fatigue	0	1 (50.0)
Vessel puncture site bruise	1 (16.7)	0
Back pain	1 (16.7)	0
Headache	2 (33.3)	1 (50.0)
Maculopapular rash	0	1 (50.0)

a AL, artemether-lumefantrine; RUX, ruxolitinib.

There were no clinically relevant changes in blood pressure, heart rate, body temperature, or respiratory rate. Postbaseline abnormal laboratory values were infrequent, none were clinically relevant, and there were no trends over time or between study groups (see Table S1 in the supplemental material). Two participants in the artemether-lumefantrine plus ruxolitinib group had a prolongation in QTcF >30 ms (+33 ms on day 8, +35 ms on day 4), but no QTcF values exceeded 450 ms (see Fig. S1 in the supplemental material).

### Pharmacokinetics of artemether, dihydroartemisinin, and lumefantrine.

**FIG 2:**
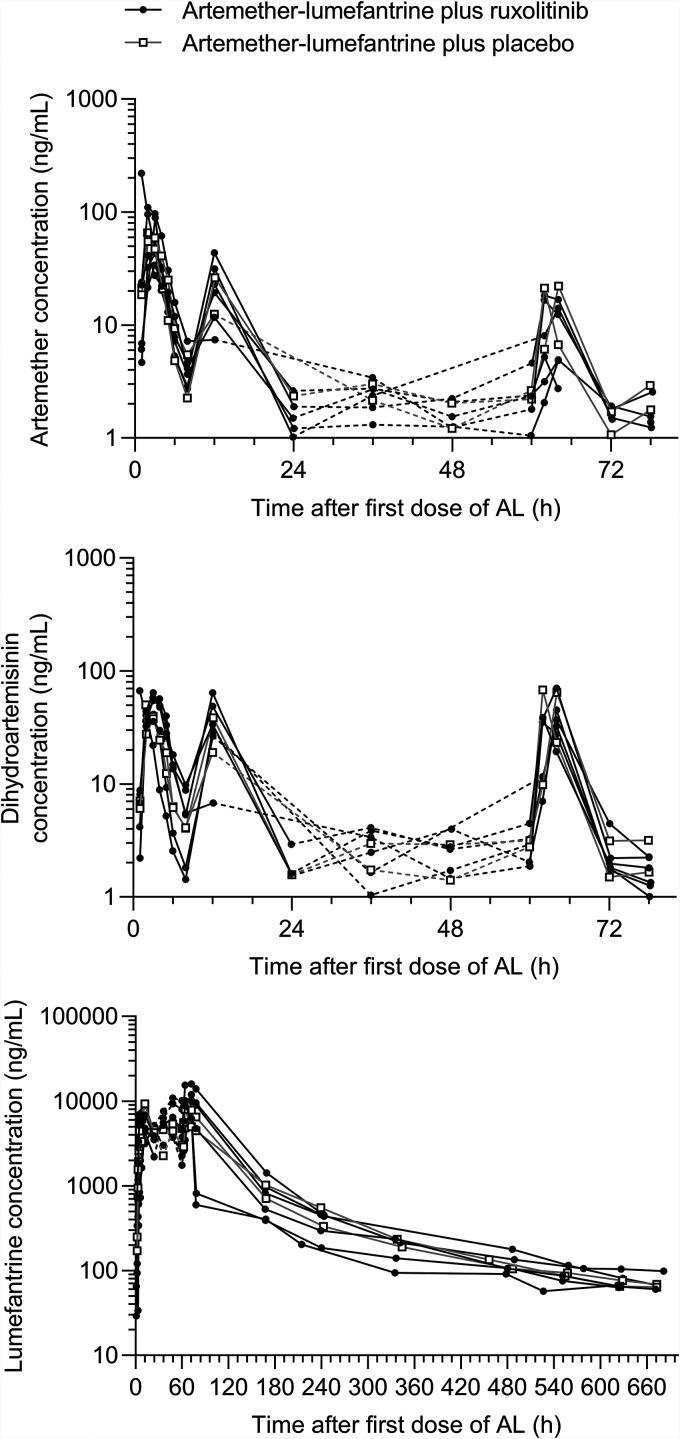
Individual participant plasma concentration-time profiles for artemether, dihydroartemisinin, and lumefantrine after coadministration with ruxolitinib or placebo. Dashed lines indicate times where sampling was sparse and do not reflect the actual drug concentrations. AL, artemether-lumefantrine.

In the artemether-lumefantrine plus placebo group, artemether was quickly absorbed with a median *T*_max_ of 2.44 h (range, 1.88 to 3.00), with a subsequent rapid decrease in artemether plasma concentrations (Fig. 2). Artemether *T*_max_ was comparable between day 1 and day 3, but *C*_max_ on day 3 was considerably lower compared to day 1 (geometric means and % coefficient of variation [CV%] = 21.6 [2.9] ng/ml versus 62.4 [7.3] ng/ml; *P* = 0.018) (Table 3; see also Table S2). The dihydroartemisinin *C*_max_ was attained at the same time on days 1 and 3 as the parent compound and also showed a rapid decline in plasma concentration. Lumefantrine exposure was 712,000 (7.4) ng·h/ml and the *t*_1/2_ was almost 200 h (Table 3). The terminal elimination phase for both artemether and dihydroartemisinin was not well characterized, and *t*_1/2_ could not be estimated.

**TABLE 3:** Pharmacokinetic parameters for artemether, dihydroartemisinin as an artemether metabolite, and lumefantrine after administration of artemether-lumefantrine with or without ruxolitinib

Analyte	Time (days)	Pharmacokinetic parameter	Mean (CV%) or median (range)^*a*^	
			AL+RUX (*n *=* *6)	AL+placebo (*n *=* *2)
Artemether	1–3	AUC_0–_*_t_* (ng·h/ml)	504 (40.5)	537 (5.0)
	1	*T*_max_ (h)	2.48 (0.98–3.05)	2.44 (1.88–3.00)
		*C*_max_ (ng/ml)	71.2 (82.7)	62.4 (7.3)
		AUC_0–8_ (ng·h/ml)	201 (54.2)	195 (14.0)
	3	*T*_max_ (h)	2.89 (1.75–4.00)	2.98 (1.92–4.03)
		*C*_max_ (ng/ml)	9.01 (72.7)	21.6 (2.9)
		AUC_0–12_ (ng·h/ml)	53.4 (67.6)	86.5 (23.1)
DHA	1–3	AUC_0–_*_t_* (ng·h/ml)	732 (11.3)	681 (13.2)
	1	*T*_max_ (h)	3.00 (0.98–3.05)	2.44 (1.88–3.00)
		*C*_max_ (ng/ml)	52.2 (25.4)	43.7 (20.0)
		AUC_0–8_ (ng·h/ml)	172 (26.6)	138 (12.3)
	3	*T*_max_ (h)	3.93 (1.75–4.00)	2.98 (1.92–4.03)
		*C*_max_ (ng/ml)	41.7 (28.5)	66.1 (3.7)
		AUC_0–12_ (ng·h/ml)	185 (27.6)	235 (10.6)
Lumefantrine	1–3	AUC_0–_*_t_* (ng·h/ml)	832,000 (23.4)	712,000 (7.4)
		AUC_0–∞_ (ng·h/ml)^*b*^	828,000 (25.3)	731,000 (6.5)
		*t*_1/2_ (h)^*b*^	196 (24.7)	197 (21.0)
	1	*T*_max_ (h)	5.98 (5.00–6.00)	6.01 (6.00–6.02)
		*C*_max_ (ng/ml)	3,510 (99.0)	5,090 (33.8)
		AUC_0–8_ (ng·h/ml)	13,100 (100.9)	19,300 (24.0)
	3	*T*_max_ (h)	12.00 (3.97–12.20)	8.02 (4.00–12.00)
		*C*_max_ (ng/ml)	10,500 (24.5)	7,890 (1.2)
		AUC_0–12_ (ng·h/ml)	93,800 (37.1)	69,500 (10.6)

a AL, artemether-lumefantrine; RUX, ruxolitinib; DHA, dihydroartemisinin. Values are geometric means (coefficient of variation percent [CV%]), except for *T*_max_, which is expressed as the median (range).

b *n *=* *5. One subject prematurely withdrew from the study after the 240-h blood sample was taken, so *t*_1/2_ and AUC_0–∞_ could not be estimated, which explains why the AUC_0–_*_t_* is larger than the AUC_0–∞_ in the artemether-lumefantrine plus ruxolitinib group.

In the artemether-lumefantrine plus ruxolitinib group, overall exposure to artemether, dihydroartemisinin and lumefantrine was consistent with the placebo group (Table 3; see also Table S3). Similar to the placebo group, the artemether *C*_max_ was lower on day 3 compared to day 1 (9.01 [72.7] ng/ml versus 71.2 [82.7] ng/ml; *P* < 0.001) (Table 3; see also Table S2). However, the artemether *C*_max_ on day 3 was lower in participants administered ruxolitinib compared to placebo (9.01 [72.7] ng/ml versus 21.6 [2.9] ng/ml; *P* = 0.021) (Table 3; see also Table S2).

### Pharmacokinetics of ruxolitinib.

**FIG 3:**
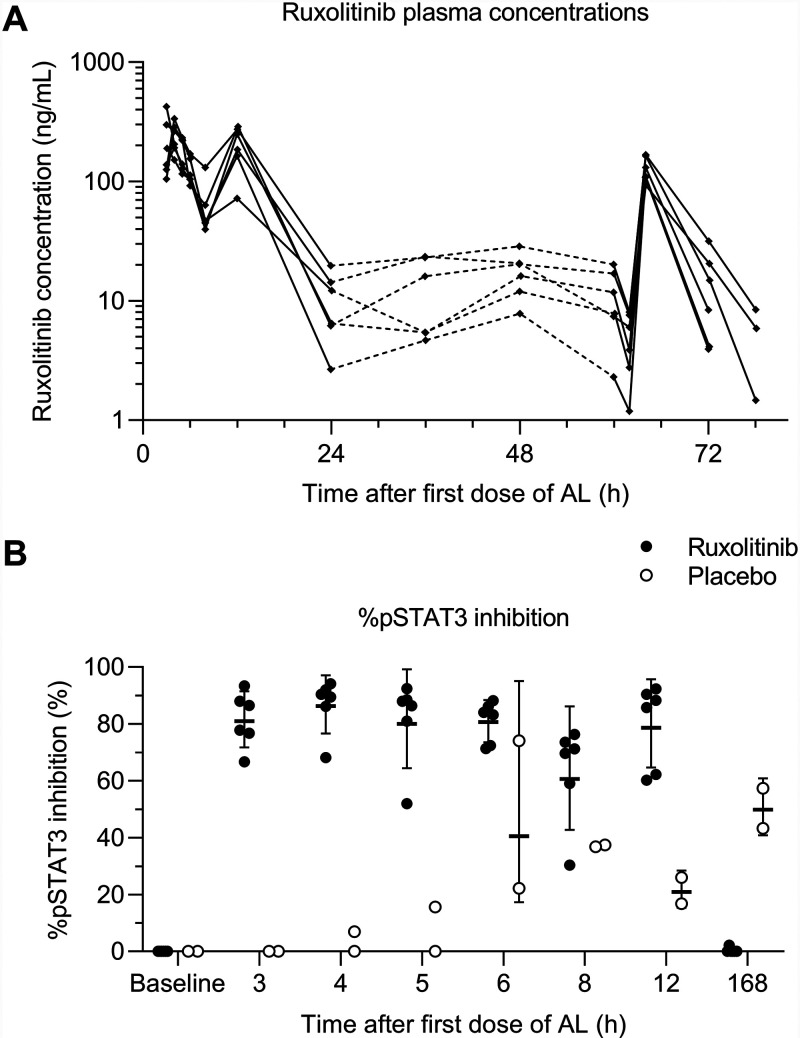
Ruxolitinib pharmacokinetics/pharmacodynamics. AL, artemether-lumefantrine. (A) Individual subject ruxolitinib plasma concentration-time profiles. Dashed lines indicate times where sampling was sparse and do not reflect the actual drug concentrations. (B) Individual %pSTAT3 inhibition. Horizontal bars indicate geometric means ± the geometric standard deviations.

Ruxolitinib mean plasma concentration increased rapidly after dosing, with a median *T*_max_ of 1.52 h (range, 0.98 to 2.00), and then rapidly decreased (Fig. 3A). The terminal elimination phase was not well characterized, and *t*_1/2_ could not be estimated. Although the ruxolitinib *t*_1/2_ could not be directly determined from concentration-time data, pharmacokinetic/pharmacodynamic model (reported below) estimates for the apparent clearance and the apparent volume of distribution for ruxolitinib were 21.8 L/h and 79.5 L, respectively, giving a half-life of 2.53 h. Although exposure to ruxolitinib on day 3 (area under the concentration-time curve from 0 to 10 h [AUC_0–10_] = 509 ng·h/ml) appeared lower compared to day 1 (AUC_0–6_ = 839 ng·h/ml; *P* = 0.005) (Table 4; see also Table S4), the day 3 blood sampling scheme was more limited than for day 1, with no blood samples taken between 2 and 10 h after the last dose of ruxolitinib, so cannot be compared. However, *C*_max_ was also lower on day 3 (126 [24.3] ng/ml) compared to day 1 (276 [37.2] ng/ml; *P* = 0.001) (Table 4; see also Table S4).

**TABLE 4:** Pharmacokinetic parameters for ruxolitinib after coadministration with artemether-lumefantrine

Time	Pharmacokinetic parameter	AL+RUX^*a*^ (*n *=* *6)
Day 1	*T*_max_ (h)	1.52 (0.98–2.0)
	*C*_max_ (ng/mL)	276 (32.7)
	AUC_0–6_ (ng·h/mL)	839 (20.8)
Day 3	*T*_max_ (h)	1.98 (1.83–2.0)
	*C*_max_ (ng/mL)	126 (24.3)
	AUC_0–10_ (ng·h/mL)	509 (34.2)

a AL, artemether-lumefantrine; RUX, ruxolitinib. Values are geometric means (coefficient of variation percent [CV%]), except for *T*_max_, which is expressed as the median (range).

### Pharmacodynamic analysis.

Examination of the %pSTAT3 inhibition versus time profiles indicated significant inhibition of pSTAT3 after administration of ruxolitinib in combination with artemether-lumefantrine compared to artemether-lumefantrine plus placebo treatment (Fig. 3B). This was supported by formal statistical comparisons of AUEC_T_; the geometric mean AUEC_T_ values were 544 ng·h/ml (CV% 15.8) for the ruxolitinib group and 181 ng·h/ml (CV% 34.4) for the placebo, giving a geometric mean ratio of 301% (90% confidence interval [CI] = 214 to 424), indicating a 3-fold greater %pSTAT3 inhibition for the ruxolitinib group compared to placebo.

### Pharmacokinetic/pharmacodynamic model.

**FIG 4:**
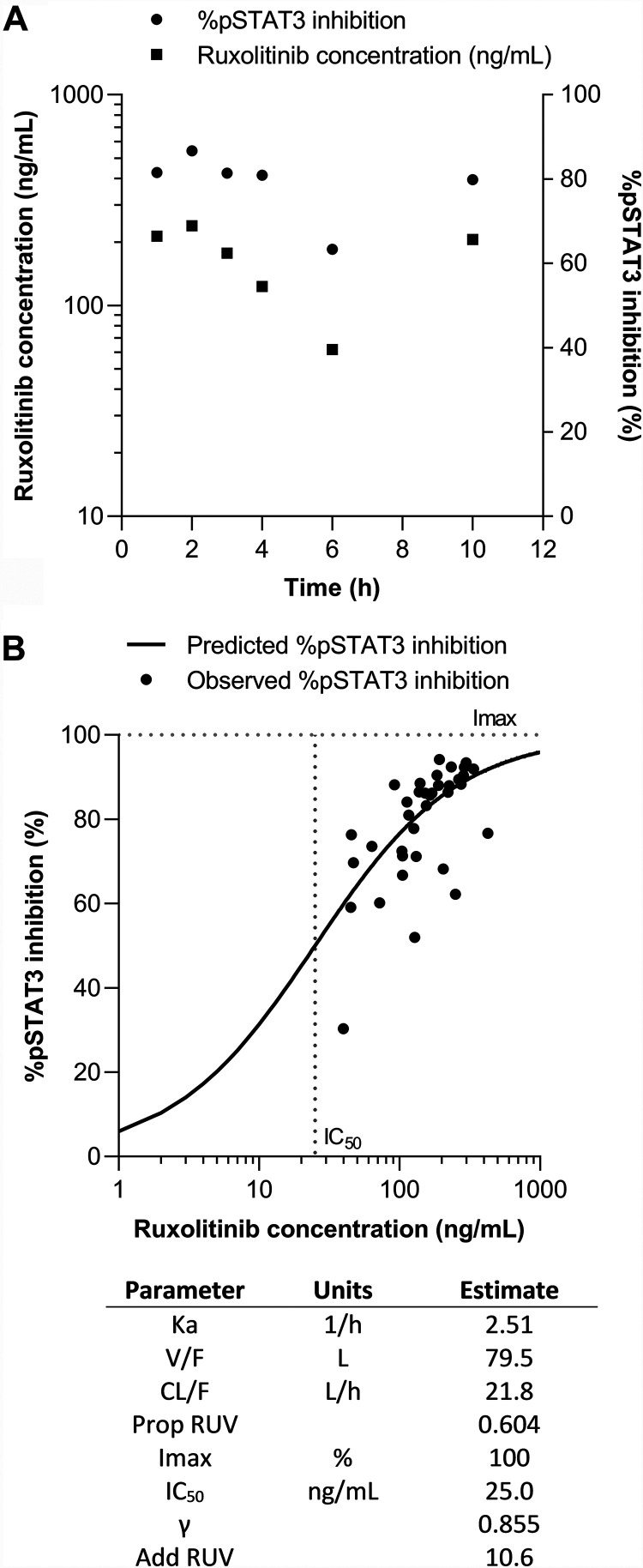
Ruxolitinib pharmacokinetic/pharmacodynamic model. (A) Mean ruxolitinib concentration and %pSTAT3 inhibition versus time. (B) Predicted and observed pharmacokinetic/pharmacodynamic relationship between ruxolitinib concentration and %pSTAT3 inhibition. Parameter abbreviations: Ka, absorption rate constant; V/F, apparent volume of distribution of the central compartment; CL/F, apparent clearance; Prop RUV, proportional residual unexplained variability Imax; IC_50_, ruxolitinib concentration at which there is 50% maximal inhibition; γ Hill coefficient; Add RUV, additive residual unexplained variability.

Based on the Akaike information criterion (36) and visual inspection of standard diagnostic plots, a one-compartment model with proportional error was selected as the most suitable model to describe ruxolitinib pharmacokinetics. Inspection of the ruxolitinib concentration and %pSTAT3 inhibition profiles showed similar time courses for pharmacokinetic and pharmacodynamic data (Fig. 4A), indicating that incorporation of a delayed effect compartment into the model was not required. This was confirmed through examination of concentration versus effect plots, indicating minimal hysteresis.

A direct effect sigmoid *E*_max_ model with additive error was selected as the most suitable model to describe the influence of ruxolitinib concentrations on %pSTAT3 inhibition. After the development of individual pharmacokinetic and pharmacodynamic models, the pharmacokinetic/pharmacodynamic relationship between ruxolitinib concentrations and %pSTAT3 inhibition was examined using a combined model for all participants administered active treatment. The results of the model fit, describing the relationship between ruxolitinib concentrations and %pSTAT3 inhibition, and are shown in Fig. 4B.

## DISCUSSION

The use of registered drugs that could promote a robust immune response to malaria infection is a novel approach aimed at preventing malaria reinfection and/or reducing the severity of clinical symptoms and progression to severe malaria. As a first step in evaluating this potential new host-directed therapeutic intervention, the safety of ruxolitinib coadministration with artemether-lumefantrine was evaluated. The dose regimen for artemether-lumefantrine was the standard adult dose for treatment of uncomplicated P. falciparum malaria (37). The ruxolitinib dose of 20 mg twice daily is the standard dose for the treatment of myelofibrosis with a platelet count >200 × 10^9^/L (38). A 3-day ruxolitinib dosing regimen was considered appropriate for this study, based on the reported safety and expected pSTAT3 inhibition of a higher dose of 25 mg twice daily over a 10-day period in healthy volunteers in a phase 1 safety trial (35).

The primary objective of this study was to assess the safety and tolerability of artemether-lumefantrine in combination with ruxolitinib. Adverse events were mild in severity, and there were no serious adverse events or adverse events considered clinically relevant or resulting in drug discontinuation or study withdrawal. It was anticipated that the short half-life of ruxolitinib would minimize the risk of adverse outcomes when coadministered with artemether-lumefantrine. Ruxolitinib coadministration did not increase the frequency of adverse events compared to placebo treatments, and there were no unexpected adverse events, considering the known safety profiles of the two study drugs (37, 38). There were no safety signals, trends, or marked differences between treatment groups in laboratory tests or vital signs in this small study.

Overall, the pharmacokinetics of ruxolitinib, artemether, dihydroartemisinin, and lumefantrine were in accordance with previously published data (37–40). The *post hoc* exploratory analysis indicated that at a 5% significance level, there were no significant differences in the pharmacokinetic parameters of artemether, dihydroartemisinin or lumefantrine measured at day 1 or day 3 between the two placebo and ruxolitinib groups, with the exception of artemether *C*_max_, which was lower on day 3 after ruxolitinib coadministration versus the placebo. Moreover, a lower exposure to ruxolitinib was observed on day 3 compared to day 1. Ruxolitinib is mainly metabolized by CYP3A4 (41), whereas artemether is metabolized by CYP3A4 and CYP2B6 and is reported to be an inducer of these drug-metabolizing enzymes (42). However, the autoinduction of artemether has been linked to CYP2B6 rather than to CYP3A4 induction. In addition, the exposure to lumefantrine as a CYP3A4 substrate was not similarly significantly decreased in this study, nor was it decreased in other studies where artemether and lumefantrine were coadministered, and so the possible role of artemether as a CYP3A4 inducer is questionable. Ruxolitinib has not been reported to induce CYP3A4 (38), and the lower artemether concentration on day 3 in the ruxolitinib group, compared to the placebo group, cannot be explained by induction of CYP3A4. Thus, the underlying mechanisms of these possible effects of ruxolitinib on artemether and artemether on ruxolitinib are currently unknown.

The pharmacodynamic profile of ruxolitinib was consistent with previous data (35), resulting in a significant 3-fold increase in inhibition of pSTAT3 activity when coadministered with artemether-lumefantrine compared to artemether-lumefantrine plus placebo. This magnitude of effect provides supporting evidence for future research exploring the potential for ruxolitinib treatment to inhibit type I IFN signaling and to disrupt the parasite-induced immune response in malaria. The ruxolitinib concentration and %pSTAT3 inhibition profiles showed similar time courses, indicating no temporal delay between drug exposure and effect. As such, the relationship between ruxolitinib concentration and %pSTAT3 inhibition was best described by a one-compartment pharmacokinetic model and a simple direct effect sigmoid *E*_max_ model. These findings support the use of ruxolitinib in combination with artemether-lumefantrine, since the pharmacodynamic effect of ruxolitinib on %pSTAT3 inhibition was retained with combination treatment.

There are some key limitations to this study. Most notably, this exploratory investigation was not a formal pharmacokinetic drug-drug interaction study. Thus, conclusions regarding the pharmacokinetics of the two drugs in combination are tentative because the study was not powered for a formal comparison. The number of participants was small, and a possible consequence of this may be the high variability in artemether (days 1 and 3) and lumefantrine (day 3) pharmacokinetic parameters when coadministered with ruxolitinib. No formal analysis of the effect of artemether-lumefantrine on ruxolitinib pharmacokinetics could be conducted, owing to the absence of a ruxolitinib plus placebo group. Also, since the blood sampling schemes on days 1 and 3 were different, comparison between the two days is difficult. This study did not evaluate the feasibility of coadministration of the artemether-lumefantrine and ruxolitinib in a clinical setting; rather, the study was designed as a preliminary analysis to confirm that there was no unexpected risk to human volunteers in subsequent clinical studies based on an unanticipated interaction. Since ruxolitinib was administered 2 h after artemether-lumefantrine, we cannot not exclude the potential for a drug-drug interaction with concurrent administration. However, the data reported here support concurrent administration in future investigations. Also, this study used a ruxolitinib dose with a known safety profile and efficacy in the human diseases for which it is indicated. However, it is unknown whether this dose would be sufficient to produce the required impact on host immunological responses to P. falciparum infection. This would require further investigation in animal models and a human VIS study.

In conclusion, ruxolitinib administered 2 h after artemether-lumefantrine was well tolerated, with adverse events consistent with the known safety profiles of the two drugs (37, 38). Ruxolitinib inhibition of pSTAT3 was demonstrated, and pharmacokinetic/pharmacodynamic modeling indicated a direct and predictable relationship between ruxolitinib plasma concentrations and %pSTAT3 inhibition. The findings of this study support further investigation of the combination of artemether-lumefantrine and ruxolitinib in healthy volunteers infected with P. falciparum.

## MATERIALS AND METHODS

### Study design and ethics.

This randomized, single-blind, placebo-controlled, single center phase 1 trial was conducted at Q-Pharm Pty, Ltd., Brisbane, Queensland, Australia, between 10 September and 17 November 2020.

The primary objective was to assess the safety and tolerability of artemether-lumefantrine plus ruxolitinib and artemether-lumefantrine plus placebo. Secondary objectives were to assess the effect of artemether-lumefantrine plus ruxolitinib or placebo on pSTAT3 inhibition and to characterize the pharmacokinetic profiles of artemether and its major metabolite dihydroartemisinin, lumefantrine, and ruxolitinib. Two initial participants (sentinel group) were recruited and randomized to either artemether-lumefantrine plus ruxolitinib or artemether-lumefantrine plus placebo. Following a safety review, a further five participants were randomized to artemether-lumefantrine plus ruxolitinib and one to artemether-lumefantrine plus placebo (Fig. 1).

The study was conducted in accordance with the clinical trial protocol, the Declaration of Helsinki (as currently revised) and the current ICH E6 Guidelines for Good Clinical Practice as adopted in Australia by the Therapeutics Good Administration. All participants provided written informed consent. The study was approved by an independent ethical review board (The Alfred Ethics Committee, Melbourne, Victoria, Australia). This study has been registered at ClinicalTrials.gov with the identifier NCT04456634. All supporting data are included in the manuscript or supplementary files, and can be requested from Medicines for Malaria Venture (mmv.org).

### Study participants.

Eligible participants were male or female healthy volunteers aged 18 to 55 years inclusive, weighing at least 50 kg with a body mass index in the range of 18 to 32 kg/m^2^. All participants had to be certified as healthy by a comprehensive clinical assessment, with normal vital signs, electrocardiogram (EGC), and laboratory assessments (hematology, clinical chemistry, and urinalysis). Pregnant and lactating women were excluded and all women of childbearing potential and males with female partners of childbearing potential had to agree to reliable contraception. Exclusion criteria were known hypersensitivity to study drugs, food/drug allergies or anaphylaxis, or a history of additional cardiac risk factors, convulsions, cancer, psychiatric illness, recurrent headache, or drug or alcohol abuse. In addition, participants could not have received any investigational drug within five half-lives or 12 weeks of the study start (whichever was longer), immunosuppressive therapy within the last year, the use of systemic anti-inflammatory drugs within the past 3 months (2 weeks for nasal/ophthalmic or topical corticosteroids), antidepressant medication within the last 12 months, any concomitant medication (except contraceptives) within 14 days or five half-lives prior to study drug administration (whichever longer), blood sampling or donation within 8 weeks prior to study drug administration, currently smoking >5 cigarettes/day, any current chronic disease, any condition that might affect drug absorption, or acute illness within 4 weeks prior to screening. Negative tests were required for pregnancy, hepatitis B, hepatitis C, human immunodeficiency virus, *Mycobacterium tuberculosis* infection (QuantiFERON-TB Gold; Qiagen, Chadstone), and drugs of abuse and alcohol.

During the study, participants were asked to refrain from alcohol consumption of more than 4 U per day and/or drugs of abuse, to not smoke more than five cigarettes a day until the conclusion of the trial, and to abstain from alcohol and smoking during clinical confinement. Participants had to refrain from excessive consumption of xanthine-containing food and beverages during the trial period, plus any consumption of such food/beverages during the confinement phase. Poppy seeds, Seville oranges, grapefruit, or grapefruit juice were to be avoided from 1 week prior to screening and for the study duration. Participants were advised to avoid strenuous exercise for 24 h and were required to fast overnight for at least 8 h before each blood collection for clinical laboratory tests.

### Study drug administration.

Four artemether-lumefantrine tablets (20/120 mg; Riamet; Novartis) were administered orally with 250 mg of full-fat milk twice daily over three consecutive days from day 1 (*t *=* *0, 8, 24, 36, 48, and 60 h) at the recommended dose for acute uncomplicated malaria (total dose, 24 tablets: 480/2,880 mg artemether-lumefantrine). Ruxolitinib (20 mg; Jakavi; Incyte) or placebo was administered orally 2 h after artemether-lumefantrine twice daily for 3 days (total dose, 120 mg ruxolitinib). The delay in ruxolitinib administration was to minimize the risk of inhibition of intestinal cytochrome P450 (CYP) 3A4 by ruxolitinib (41), which could potentially lead to increased exposure of artemether and lumefantrine, since both drugs are CYP3A4 substrates (41), and decreased exposure to the active metabolite of the artemether, dihydroartemisinin. All study drug administrations were observed by clinical staff.

### Study procedures.

Screening was conducted within 28 days prior to administration of the first dose of study drug. Participants were confined to the study center from day −1 until day 4, with scheduled outpatient follow-up visits on days 8, 11, 15, 21, 24, and 27 and the end-of-study visit on day 29 (Fig. 1).

A physical examination was performed at screening, day −1, and day 29; a medical history was taken at screening, day −1, and before drug administration. Urine drug testing and alcohol breath testing were performed at screening and day −1. Vital signs, temperature, and respiration rate were noted at screening, day −1, predose on day 1, follow-up visits, and day 29. Standard single 12-lead ECGs were recorded at screening; on day −1 within 60 min prior to the first dose of artemether-lumefantrine; at 24, 48, and 72 h after the first dose; and on days 8, 15, and 29. Samples were collected for clinical chemistry, urinalysis, and hematology at screening; at day −1; predose on day 1; at 24, 48, and 72 h after the first artemether-lumefantrine dose; and at days 8, 15, and 29. All blood samples were collected either by direct venipuncture or indwelling cannula. Adverse events were assessed at all visits.

### Adverse events and laboratory assessments.

Safety endpoints were the frequency of adverse events and serious adverse events, and abnormal vital signs, 12-lead ECG, hematology, clinical biochemistry, coagulation, urinalysis, and physical examination. Adverse events were reported by system organ class and preferred term, according to the Medical Dictionary for Regulatory Activities (MedDRA, version 23.1). Serious adverse events were defined as those that were life-threatening, resulted in disability, hospitalization, or death. Adverse event severity was graded using the Common Terminology Criteria for Adverse Events (CTCAE, version 5.0, 2017). Adverse events were reported using descriptive statistics. Laboratory test values outside the normal range were reported and were considered clinically relevant if they were at least grade 2 in severity, if they occurred with associated symptoms, or if they required intervention.

### Pharmacokinetics.

Blood samples for measurement of drug blood levels were collected predose and then on day 1 after the first artemether-lumefantrine administration (*t *=* *0) at *t = *1, 2, 3, 4, 5, 6, 8, 12, 24, 36, 48, 60, 62, 64, 72, and 78 h and at days 8, 11, 15, 21, 24, 27, and 29 (*t *=* *168, 240, 336, 480, 552, 624, and 672 h postdose, respectively). For time points coinciding with drug administration, blood sampling occurred prior to study drug administration.

Artemether, dihydroartemisinin, lumefantrine, and ruxolitinib plasma concentrations were measured using validated liquid chromatography-tandem mass spectrometry methods at Swiss BioQuant AG, Reinach, Switzerland. Linear ranges were 1.00 to 500 ng/ml for artemether and dihydroartemisinin, 10.0 to 10,000 ng/ml for lumefantrine, and 1.00 to 1,000 ng/ml for ruxolitinib. The precision values (%CV) of the assay quality control samples were 2.7 to 5.1% for artemether, 3.1 to 4.8% for dihydroartemisinin, 5.7 to 11.4% for lumefantrine, and 0.9 to 2.1% for ruxolitinib. The accuracies (bias) of the assays for low- to high-quality control concentrations were −9.7 to 0.5% for artemether, −6.7 to 0.8% for dihydroartemisinin, −6.0 to 2.5% for lumefantrine, and −3.0 to 0.8% for ruxolitinib.

Pharmacokinetic parameters were estimated from observed plasma concentration-time data using noncompartmental methods as follows: maximum observed plasma concentration (*C*_max_), time to *C*_max_ (*T*_max_), apparent terminal-phase half-life (*t*_1/2_), and the AUCs, including AUC from time zero (i.e., predose on day 1) until time *t* of the last quantifiable concentration after the final study drug administration on day 3 (AUC_0–_*_t_*), the predicted AUC from time zero to infinity (AUC_0–∞_), and the AUC from predose to 6 or 8 h postdose on day 1 (AUC_0–6_ and AUC_0–8_) and from predose to 10 or 12 h postdose on day 3 (AUC_0–10_ and AUC_0–12_). Pharmacokinetic analysis was conducted using Phoenix WinNonlin (version 8.2; Pharsight Corporation).

### Ruxolitinib pharmacokinetic/pharmacodynamic analysis.

Blood samples for pSTAT3 evaluations were collected at predose and then after the first dose of ruxolitinib at *t* = −2, −1, 0, 2, 3, 4, 6, 10, and 166 h (i.e., relative to artemether-lumefantrine dosing at *t *=* *0, 1, 2, 3, 4, 5, 6, 8, 12, and 168 h). Blood samples were stimulated with 100 ng/ml of human IL-6 for 15 min, and the white blood cell fraction isolated after red blood cell lysis. *Ex vivo* pSTAT3 inhibition was assessed in the white blood cell fraction using Meso Scale Discovery immunoassay kits for measurement of pSTAT3 at TetraQ, Brisbane, Australia. The relative pSTAT3 levels were normalized to total STAT3, as previously described (35). The percent pSTAT3 (%pSTAT3) inhibition was determined for each sampling time point and was calculated as follows:

$$\text{\%pSTAT3 inhibition }=100\;\times \;\frac{\text{pSTAT}3_{0}\;\;-\;{\text{ pSTAT3}}_{i}}{{\text{pSTAT3}}_{0}},$$

where pSTAT3_0_ was the pSTAT3 level prior to treatment initiation (i.e., the average of pSTAT3 levels at −2, −1, and 0 h relative to ruxolitinib dosing) and pSTAT3_i_ was the pSTAT3 level at the i*th* time point. Negative calculated %pSTAT3 inhibition values were assigned a value of 0%. Ruxolitinib concentrations below the lower limit of quantification (1 ng/ml) were handled using the M3 method (43).

For each participant, %pSTAT3 inhibition data were used for the calculation of the area under the pharmacodynamic effect versus time profile over the ruxolitinib/placebo dosing interval on day 1 (AUEC_T_), calculated using the linear trapezoidal method. The pharmacokinetic/pharmacodynamic relationship between ruxolitinib concentration and %pSTAT3 inhibition was calculated using sigmoidal curve fitting according to the following equation:

$$I\;=\;\frac{I_{\text{max}}\;\times \;C^{\gamma }}{C^{\gamma }\;+\;{\text{IC}}_{50}{}^{\gamma }},$$

where *I* is the %pSTAT3 inhibition, *C* is the ruxolitinib concentration, *I*_max_ is the theoretical maximum %pSTAT3 inhibition, IC_50_ is the ruxolitinib concentration at which there is 50% maximal inhibition, and γ is the Hill coefficient.

ANOVA was used to perform treatment comparisons of log_e_-transformed AUEC_T_ data (LnAUEC_T_). The residual error (error mean square) was used to construct the 90% confidence intervals (CIs) for the ratio of treatment means. No statistical difference was concluded if the 90% CIs were within the standard regulatory limits of 80 to 125%.

The relationship between ruxolitinib concentrations and %pSTAT3 inhibition was examined through the development of a pharmacokinetic/pharmacodynamic model. One- and two-compartment models with first-order absorption and elimination from the central compartment were explored, including models incorporating absorption lag time. Pharmacokinetic/pharmacodynamic analysis was conducted using Phoenix WinNonlin (version 8.2; Pharsight Corporation).

### Sample size.

Since the combination of artemether-lumefantrine and ruxolitinib has not been previously tested, a first-in-human approach was adopted with a sample size of eight participants.

### *Post hoc* analysis.

The study was primarily a safety assessment and not powered to detect differences in pharmacokinetics between the two treatment groups. However, since apparent differences were noted in artemether pharmacokinetics between days 1 and 3 and between the ruxolitinib and placebo groups, an exploratory *post hoc* statistical comparison was conducted using the Kruskal-Wallis test for *T*_max_ parameters and a two-sample *t* test for the change in log_10_-transformed *C*_max_ and AUC parameters. Paired *t* tests and Wilcoxon rank tests were used to assess differences over time. All statistical analyses were two-sided tests and were performed in STATA version 15.1. The significance was set at an α-level of 0.05.
