# Supplementary material for: Safety, Tolerability, Pharmacokinetics, and Pharmacodynamics of Coadministered Ruxolitinib and Artemether-Lumefantrine in Healthy Adults
Source: Antimicrob Agents Chemother. 2022 Jan 18;66(1):e01584-21. doi: 10.1128/AAC.01584-21 (PMC8765294; doi:10.1128/AAC.01584-21)
Supplement: Supplemental file 1 — Supplemental figure and tables. Download AAC.01584-21-s0001.pdf, PDF file, 0.8 MB [file aac.01584-21-s0001.pdf]

Supplementary material for

Safety, Tolerability, Pharmacokinetics and Pharmacodynamics of Co-administered Ruxolitinib and Artemether-Lumefantrine in Healthy Adults

M. Farouk Chughlay,[a] # Karen I. Barnes,[b] Myriam El Gaaloul,[a] Nada Abla,[a] Jörg J Möhrle,[a] Paul Griffin,[c,d,e,f] Paul van Giersbergen,[g] Stephanie E Reuter,[h] Hayley B. Schultz,[h] Anita Kress,[i] Peter Tapley,[j] Rebecca A. Webster,[c] Timothy Wells,[a] James S. McCarthy,[c,e] Bridget E. Barber,[c] Louise Marquart,[c] Michelle J. Boyle,[c] Christian R. Engwerda,[c] Stephan Chalon[a]

[a]Medicines for Malaria Venture, Geneva, Switzerland

[b]Division of Clinical Pharmacology, Department of Medicine, University of Cape Town, Cape Town, South Africa

[c]QIMR Berghofer Medical Research Institute, Brisbane, Queensland Australia

[d]Q-Pharm Pty Ltd, Herston, Queensland, Australia

[e]University of Queensland, Brisbane, Queensland, Australia

[f]Mater Health Services, South Brisbane, Queensland, Australia

[g]Van Giersbergen Consulting, Wuenheim, France

[h]UniSA Clinical and Health Sciences, University of South Australia, Adelaide, South Australia, Australia

[i]Swiss BioQuant, Reinach, Switzerland

[j]TetraQ, Brisbane, Queensland, Australia

#Corresponding author.

Contents

Table S1 Out of normal range values for hematology, clinical chemistry, and urinalysis relative to screening and baseline (day −1)……………………………………………………………………3

Figure S1 Individual participant values for Fridericia-corrected QT (QTcF)…………………………6

Table S2 Post-hoc analysis comparing the change over time in T_{max} and C_{max} parameters within each treatment group assessed using Wilcoxon signed-rank test for T_{max} or paired t-test of the log_{10} transformed C_{max} parameter, and summarized either as median (range) for T_{max} or as the ratio of day 3 relative to day 1 presented as a percent and 95% confidence interval for C_{max} (relative change)……………………………………………………………………………………7

Table S3 Post-hoc analysis comparing pharmacokinetic parameters for artemether, dihydroartemisinin as an artemether metabolite, and lumefantrine following administration of artemether-lumefantrine with or without ruxolitinib. Differences between treatment groups were assessed using either the Kruskal-Wallis for T_{max}, or two-sample t-test for log_{10}-transformed C_{max}, AUC, and half-life parameters………………………………………………………………..8

Table S4 Post-hoc analysis comparing ruxolitinib pharmacokinetic parameters for the artemether-lumefantrine plus ruxolitinib group (*n* = 6) between day 1 and day 3. Differences over the two time-points were compared non-parametrically using Wilcoxon signed-rank test, and differences of the log_{10} parameters were compared using a paired t-test and summarized as the ratio of day 3 relative to day 1 and presented as a percent and 95% confidence interval……10

Table S1 Out of normal range values for hematology, clinical chemistry, and urinalysis relative to screening and baseline (day −1).

Parameter (unit)	Visit	Artemether-lumefantrine plus ruxolitinib (*n* = 6)									Artemether-lumefantrine plus placebo (*n* = 2)								
		n	**Absolute values**					**Reference range, *n* (%)**			***n***	**Absolute values**					**Reference range, *n* (%)**		
			**Mean**	**SD**	**Median**	**Min**	**Max**	**Low**	**Normal**	**High**		**Mean**	**SD**	**Median**	**Min**	**Max**	**Low**	**Normal**	**High**
Eosinophils (10^9/L)	Screening	6	0.150	0.105	0.144	0.03	0.43	1 (16.7)	5 (83.3)	0	2	0.260	0.260	0.226	0.10	0.42	0	2 (100)	0
	Day −1	6	0.088	0.070	0.077	0.02	0.24	1 (16.7)	5 (83.3)	0	2	0.300	0.300	0.198	0.16	0.44	0	2 (100)	0
	Day 2	6	0.103	0.090	0.062	0.03	0.19	1 (16.7)	5 (83.3)	0	2	0.310	0.310	0.283	0.11	0.51	0	1 (50.0)	1 (50.0)
	Day 3	6	0.100	0.110	0.047	0.03	0.15	1 (16.7)	5 (83.3)	0	2	0.305	0.305	0.219	0.15	0.46	0	2 (100)	0
	Day 8	6	0.135	0.110	0.117	0.01	0.35	1 (16.7)	5 (83.3)	0	2	0.325	0.325	0.290	0.12	0.53	0	1 (50.0)	1 (50.0)
	Day 29	5	0.176	0.130	0.162	0.03	0.44	1 (16.7)	4 (66.7)	0	2	0.320	0.320	0.226	0.16	0.48	0	2 (100)	0
Hemoglobin (g/L)	Screening	6	141.3	139.5	6.9	135	153	0	6 (100)	0	2	142.0	142.0	14.1	132	152	0	2 (100)	0
	Day −1	6	142.0	142.5	7.8	133	155	0	6 (100)	0	2	143.0	143.0	12.7	134	152	0	2 (100)	0
	Day 8	6	134.3	134.5	12.4	113	149	1 (16.7)	5 (83.3)	0	2	143.0	143.0	12.7	134	152	0	2 (100)	0
Lymphocytes (10^9/L)	Screening	6	2.345	1.945	0.912	1.67	4.04	0	5 (83.3)	1 (16.7)	2	2.310	2.310	0.721	1.80	2.82	0	2 (100)	0
	Day −1	6	2.498	2.445	0.874	1.55	3.88	0	6 (100)	0	2	2.390	2.390	0.820	1.81	2.97	0	2 (100)	0
	Day 15	4	2.633	2.260	1.119	1.74	4.27	0	3 (50.0)	1 (16.7)	2	1.810	1.810	0.184	1.68	1.94	0	2 (100)	0
Neutrophils (10^9/L)	Screening	6	4.735	5.385	1.474	1.87	5.82	0	6 (100)	0	2	5.025	5.025	3.415	2.61	7.44	0	2 (100)	0
	Day −1	6	5.005	5.060	1.074	3.09	6.20	0	6 (100)	0	2	6.555	6.555	0.346	6.31	6.80	0	2 (100)	0
	Day 2	6	3.303	2.855	1.330	1.77	5.09	1 (16.7)	5 (83.3)	0	2	3.960	3.960	1.259	3.07	4.85	0	2 (100)	0
	Day 3	6	2.590	2.265	1.208	1.42	4.93	1 (16.7)	5 (83.3)	0	2	3.375	3.375	0.856	2.77	3.98	0	2 (100)	0
Erythrocytes (10^{12}/L)	Screening	6	4.562	4.525	0.364	4.18	5.20	0	6 (100)	0	2	4.600	4.600	0.495	4.25	4.95	0	2 (100)	0
	Day −1	6	4.602	4.565	0.310	4.17	4.95	0	6 (100)	0	2	4.620	4.620	0.339	4.38	4.86	0	2 (100)	0
	Day 8	6	4.350	4.335	0.447	3.75	5.05	1 (16.7)	5 (83.3)	0	2	4.635	4.635	0.375	4.37	4.90	0	2 (100)	0
Reticulocytes (10^9/L)	Screening	6	58.95	56.70	18.30	39.3	92.6	0	6 (100)	0	2	69.25	69.25	9.55	62.5	76.0	0	2 (100)	0
	Day −1	6	57.28	52.95	13.12	47.5	82.7	0	6 (100)	0	2	66.95	66.95	9.83	60.0	73.9	0	2 (100)	0
	Day 8	6	60.92	59.45	22.42	26.3	91.9	0	6 (100)	0	2	85.95	85.95	23.26	69.5	102.4	0	1 (50.0)	1 (50.0)
Leukocytes (10^9/L)	Screening	6	7.90	8.35	1.88	4.3	9.5	0	6 (100)	0	2	8.15	8.15	4.03	5.3	11.0	0	2 (100)	0
	Day −1	6	8.22	8.15	1.41	6.6	9.8	0	6 (100)	0	2	9.95	9.95	0.35	9.7	10.2	0	2 (100)	0
	Day 8	6	6.73	7.05	1.87	3.9	8.7	1 (16.7)	5 (83.3)	0	2	6.65	6.65	1.06	5.9	7.4	0	2 (100)	0
Albumin (g/L)	Screening	6	46.7	46.5	4.0	43	54	0	5 (83.3)	1 (16.7)	2	40.0	40.0	2.8	38	42	0	2 (100)	0
	Day −1	6	43.3	42.5	3.4	40	50	0	5 (83.3)	1 (16.7)	2	42.0	42.0	4.2	39	45	0	2 (100)	0
	Day 2	6	40.0	40.0	0.9	39	41	0	6 (100)	0	2	37.0	37.0	9.9	30	44	1 (50.0)	1 (50.0)	0

Parameter (unit)	Visit	Artemether-lumefantrine plus ruxolitinib (n = 6)									Artemether-lumefantrine plus placebo (n = 2)								
		n	Absolute values					Reference range, *n* (%)			*n*	Absolute values					Reference range, *n* (%)		
			Mean	SD	Median	Min	Max	Low	Normal	High		Mean	SD	Median	Min	Max	Low	Normal	High
	Day 4	6	42.0	41.0	3.1	39	48	0	5 (83.3)	1 (16.7)	2	40.0	40.0	1.4	39	41	0	2 (100)	0
	Day 29	5	41.6	41.0	3.7	39	48	0	4 (66.7)	1 (16.7)	2	41.0	41.0	2.8	39	43	0	2 (100)	0
Alkaline phosphatase (U/L)	Screening	6	61.3	64.5	8.8	49	71	0	6 (100)	0	2	46.5	46.5	2.1	45	48	0	2 (100)	0
	Day −1	6	55.2	57.0	9.6	42	66	0	6 (100)	0	2	51.5	51.5	9.2	45	58	0	2 (100)	0
	Day 2	6	48.0	51.5	8.5	33	56	1 (16.7)	5 (83.3)	0	2	41.5	41.5	20.5	27	56	1 (50.0)	1 (50.0)	0
	Day 4	6	47.7	51.0	9.7	31	57	1 (16.7)	5 (83.3)	0	2	50.0	50.0	1.4	49	51	0	2 (100)	0
Bicarbonate (mmol/L)	Screening	6	27.7	27.5	2.7	25	32	0	6 (100)	0	2	28.5	28.5	3.5	26	31	0	2 (100)	0
	Day −1	6	28.5	28.0	2.2	26	32	0	6 (100)	0	2	28.5	28.5	3.5	26	31	0	2 (100)	0
	Day 2	6	26.3	27.0	2.5	23	30	0	6 (100)	0	2	25.0	25.0	7.1	20	30	1 (50.0)	1 (50.0)	0
	Day 8	6	27.3	26.5	2.9	25	33	0	5 (83.3)	1 (16.7)	2	29.5	29.5	3.5	27	32	0	2 (100)	0
Direct bilirubin (µmol/L)	Screening	6	2.5	2.5	0.5	2	3	0	6 (100)	0	2	3.0	3.0	1.4	2	4	0	2 (100)	0
	Day −1	6	1.5	1.5	1.4	0	3	0	6 (100)	0	2	2.0	2.0	0.0	2	2	0	2 (100)	0
	Day 3	6	2.2	0.0	4.0	0	10	0	5 (83.3)	1 (16.7)	2	1.5	1.5	0.7	1	2	0	2 (100)	0
Calcium (mmol/L)	Screening	6	2.432	2.435	0.063	2.36	2.54	0	6 (100)	0	2	2.320	2.320	0.042	2.29	2.35	0	2 (100)	0
	Day −1	6	2.393	2.380	0.069	2.33	2.52	0	6 (100)	0	2	2.360	2.360	0.057	2.32	2.40	0	2 (100)	0
	Day 2	6	2.288	2.270	0.043	2.26	2.37	0	6 (100)	0	2	2.160	2.160	0.283	1.96	2.36	1 (50.0)	1 (50.0)	0
Creatine kinase (U/L)	Screening	6	83.3	78.0	23.1	61	128	0	6 (100)	0	2	76.5	76.5	6.4	72	81	0	2 (100)	0
	Day −1	6	92.7	74.5	46.8	49	175	0	6 (100)	0	2	78.5	78.5	9.2	72	85	0	2 (100)	0
	Day 8	6	159.3	136.5	96.1	70	335	0	4 (66.7)	2 (33.3)	2	78.0	78.0	21.2	63	93	0	2 (100)	0
	Day 15	4	118.0	117.0	46.1	74	164	0	3 (50.0)	1 (16.7)	2	77.0	77.0	18.4	64	90	0	2 (100)	0
	Day 29	5	118.0	88.0	60.2	70	214	0	4 (66.7)	1 (16.7)	2	81.5	81.5	12.0	73	90	0	2 (100)	0
Chloride (mmol/L)	Screening	6	101.7	101.5	1.2	100	103	0	6 (100)	0	2	103.0	103.0	1.4	102	104	0	2 (100)	0
	Day −1	6	102.7	103.0	2.3	100	105	0	6 (100)	0	2	102.5	102.5	2.1	101	104	0	2 (100)	0
	Day 2	6	104.5	105.0	1.6	102	106	0	6 (100)	0	2	107.0	107.0	7.1	102	112	0	1 (50.0)	1 (50.0)
Glucose (mmol/L)	Screening	6	4.12	4.10	0.48	3.5	4.7	0	6 (100)	0	2	4.10	4.10	0.42	3.8	4.4	0	2 (100)	0
	Day −1	6	4.28	4.30	0.43	3.6	4.7	0	6 (100)	0	2	4.20	4.20	0.00	4.2	4.2	0	2 (100)	0
	Day 29	5	4.86	4.40	1.18	3.9	6.9	0	4 (66.7)	1 (16.7)	2	4.80	4.80	0.85	4.2	5.4	0	2 (100)	0
Potassium (mmol/L)	Screening	6	4.10	4.00	0.28	3.9	4.6	0	6 (100)	0	2	4.25	4.25	0.07	4.2	4.3	0	2 (100)	0
	Day −1	6	4.30	4.30	0.35	3.8	4.8	0	6 (100)	0	2	4.15	4.15	0.07	4.1	4.2	0	2 (100)	0
	Day 3	6	4.45	4.30	0.48	4.1	5.4	0	5 (83.3)	1 (16.7)	2	4.10	4.10	0.28	3.9	4.3	0	2 (100)	0

Parameter (unit)	Visit	Artemether-lumefantrine plus ruxolitinib (*n* = 6)									Artemether-lumefantrine plus placebo (*n* = 2)								
		n	Absolute values					Reference range, *n* (%)			*n*	Absolute values					Reference range, *n* (%)		
			Mean	SD	Median	Min	Max	Low	Normal	High		Mean	SD	Median	Min	Max	Low	Normal	High
Lactate dehydrogenase (U/L)	Screening	6	146.7	151.0	16.1	119	161	1 (16.7)	5 (83.3)	0	2	156.0	156.0	5.7	152	160	0	2 (100)	0
	Day −1	6	140.2	141.0	15.5	113	156	1 (16.7)	5 (83.3)	0	2	160.0	160.0	22.6	144	176	0	2 (100)	0
	Day 2	6	137.5	132.0	25.2	116	184	2 (33.3)	4 (66.7)	0	2	183.0	183.0	29.7	162	204	0	2 (100)	0
	Day 4	6	133.2	132.0	18.6	114	162	2 (33.3)	4 (66.7)	0	2	138.5	138.5	0.7	138	139	0	2 (100)	0
LDL cholesterol (mmol/L)	Screening	6	2.23	2.35	0.78	1.0	3.3	1 (16.7)	4 (66.7)	1 (16.7)	2	2.90	2.90	0.00	2.9	2.9	0	2 (100)	0
Magnesium (mmol/L)	Screening	6	0.82	0.80	0.08	0.7	0.9	0	6 (100)	0	2	0.80	0.80	0.00	0.8	0.8	0	2 (100)	0
	Day −1	6	0.78	0.80	0.04	0.7	0.8	0	6 (100)	0	2	0.75	0.75	0.07	0.7	0.8	0	2 (100)	0
	Day 3	6	0.75	0.80	0.08	0.6	0.8	1 (16.7)	5 (83.3)	0	2	0.75	0.75	0.07	0.7	0.8	0	2 (100)	0
Phosphate (mmol/L)	Screening	6	1.218	1.180	0.118	1.07	1.38	0	6 (100)	0	2	1.140	1.140	0.071	1.09	1.19	0	2 (100)	0
	Day −1	6	1.125	1.135	0.160	0.94	1.33	0	6 (100)	0	2	1.230	1.230	0.028	1.21	1.25	0	2 (100)	0
	Day 8	6	1.158	1.220	0.195	0.85	1.35	1 (16.7)	5 (83.3)	0	2	1.105	1.105	0.177	0.98	1.23	0	2 (100)	0
Protein (g/L)	Screening	6	77.5	77.5	3.7	73	84	0	6 (100)	0	2	71.5	71.5	6.4	67	76	0	2 (100)	0
	Day −1	6	73.0	72.5	4.3	68	81	0	6 (100)	0	2	72.5	72.5	6.4	68	77	0	2 (100)	0
	Day 2	6	68.0	68.0	1.1	66	69	0	6 (100)	0	2	66.0	66.0	12.7	57	75	1 (50.0)	1 (50.0)	0
	Day 3	6	69.3	69.5	4.2	63	75	1 (16.7)	5 (83.3)	0	2	68.0	68.0	0.0	68	68	0	2 (100)	0
Sodium (mmol/L)	Screening	6	137.7	137.0	2.5	135	142	0	6 (100)	0	2	137.5	137.5	3.5	135	140	0	2 (100)	0
	Day −1	6	137.2	138.0	2.7	133	140	1 (16.7)	5 (83.3)	0	2	136.5	136.5	0.7	136	137	0	2 (100)	0
	Day 29	5	139.0	140.0	3.2	134	142	1 (16.7)	4 (66.7)	0	2	139.0	139.0	2.8	137	141	0	2 (100)	0
Urinary pH	Screening	6	6.75	6.75	0.69	6.0	7.5	0	6 (100)	0	2	6.50	6.50	1.41	5.5	7.5	0	2 (100)	0
	Day −1	6	7.08	7.00	1.07	6.0	8.5	0	5 (83.3)	1 (16.7)	2	6.00	6.00	0.71	5.5	6.5	0	2 (100)	0
Urinary protein/ creatinine (mg/mol)	Screening	6	12.2	10.0	3.7	10	19	0	6 (100)	0	2	11.0	11.0	1.4	10	12	0	2 (100)	0
	Day −1	6	12.8	10.0	5.6	10	24	0	6 (100)	0	2	11.0	11.0	1.4	10	12	0	2 (100)	0
	Day 8	6	16.7	10.0	11.5	10	38	0	5 (83.3)	1 (16.7)	2	10.0	10.0	0.0	10	10	0	2 (100)	0

Figure S1 Individual participant values for Fridericia-corrected QT (QTcF).

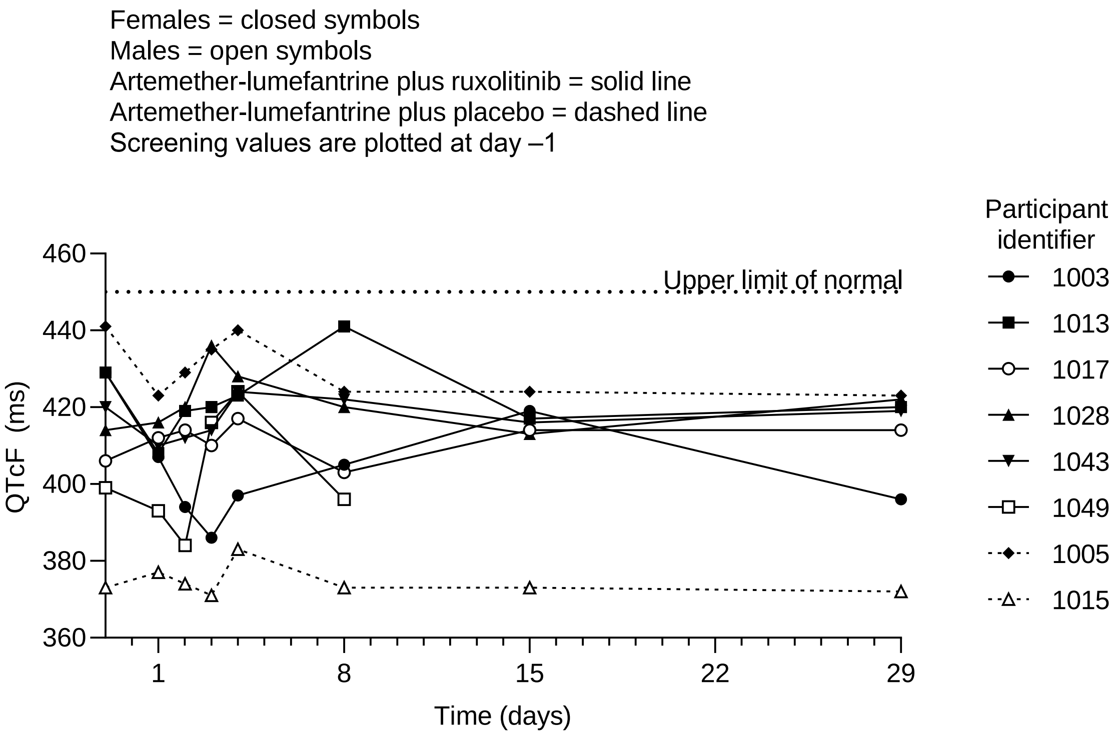

Table S2 Post-hoc analysis comparing the change over time in T_{max} and C_{max} parameters within each treatment group assessed using Wilcoxon signed-rank test for T_{max} or paired t-test of the $\log_{10}$ transformed C_{max} parameter, and summarized either as median (range) for T_{max} or as the ratio of day 3 relative to day 1 presented as a percent and 95% confidence interval for C_{max} (relative change).

**Analyte**	**Parameter**[a]	**Artemether-lumefantrine plus**	**Day 1**	**Day 3**	**Relative change**[b] **(95% CI)**	**Test statistic**	**p value**
Artemether	C_{max}	Placebo (n = 2)	62.4 (7.3)	21.6 (2.9)	34.7 (23.5, 51.1)	$t_1 = -34.8$	0.018
		Ruxolitinib (n = 6)	71.2 (82.7)	9.01 (72.7)	12.6 (9.3, 17.1)	$t_5 = -17.5$	<0.001
	T_{max}	Placebo (n = 2)	2.44 (1.88–3.00)	2.98 (1.92–4.03)	0.5 (−1.1, 2.2)	Z = 0.45	0.65
		Ruxolitinib (n = 6)	2.48 (0.98–3.05)	2.89 (1.75–4.00)	0.8 (−0.1, 1.0)	Z = 1.57	0.12
Dihydroartemisinin	C_{max}	Placebo (n = 2)	43.7 (20.0)	66.1 (3.7)	151 (18, 1260)	$t_1 = 2.48$	0.24
		Ruxolitinib (n = 6)	52.2 (25.4)	41.7 (28.5)	79.9 (59.4, 107.4)	$t_5 = -1.95$	0.11
	T_{max}	Placebo (n = 2)	2.44 (1.88–3.00)	2.98 (1.92–4.03)	0.5 (−1.1, 2.2)	Z = 0.45	0.65
		Ruxolitinib (n = 6)	3.00 (0.98–3.05)	3.93 (1.75–4.00)	0.9 (−1.1, 1.0)	Z = 0.95	0.34
Lumefantrine	C_{max}	Placebo (n = 2)	5,090 (33.8)	7,890 (1.2)	155 (9, 2693)	$t_1 = 1.96$	0.30
		Ruxolitinib (n = 6)	3,510 (99.0)	10,500 (24.5)	300 (128, 704)	$t_5 = 3.31$	0.021
	T_{max}	Placebo (n = 2)	6.01 (6.00–6.02)	8.02 (4.00–12.00)	2.0 (−2.0, 6.0)	Z = 0.45	0.65
		Ruxolitinib (n = 6)	5.98 (5.00–6.00)	12.00 (3.97–12.20)	6.0 (−1.0, 7.2)	Z = 1.99	0.046

[a] C_{max} (ng/mL), geometric mean (%CV) and T_{max} (h), median (range)

[b] Change of day 3 pharmacokinetic parameter estimates compared to day 1, presented either as the median and range of the difference between day 3 and day 1 for T_{max} parameters; or presented as the percentage change and 95% CI of pharmacokinetic parameter measured at day 3 relative to day 1.

Table S3 Post-hoc analysis comparing pharmacokinetic parameters for artemether, dihydroartemisinin as an artemether metabolite, and lumefantrine following administration of artemether-lumefantrine with or without ruxolitinib. Differences between treatment groups were assessed using either the Kruskal-Wallis for T_{max}, or two-sample t-test for $\log_{10}$-transformed C_{max}, AUC, and half-life parameters.

Analyte	Time	Pharmacokinetic parameter	Artemether-lumefantrine plus ruxolitinib (*n* = 6)	Artemether-lumefantrine plus placebo (*n* = 2)	Test statistic	p value
Artemether	Days 1–3	$AUC_{0–t}$ (ng·h/mL)	504 (40.5)	537 (5.0)	$t_{5.44} = -0.40$	0.70[a]
	Day 1	T_{max} (h)	2.48 (0.98–3.05)	2.44 (1.88–3.00)	$\chi_1^2 = 0.028$	0.87
		C_{max} (ng/mL)	71.2 (82.7)	62.4 (7.3)	$t_6 = 0.24$	0.82
		$AUC_{0–8h}$ (ng·h/mL)	201 (54.2)	195 (14.0)	$t_6 = 0.08$	0.94
	Day 3	T_{max} (h)	2.89 (1.75–4.00)	2.98 (1.92–4.03)	$\chi_1^2 = 0.44$	0.51
		C_{max} (ng/mL)	9.01 (72.7)	21.6 (2.9)	$t_{5.06} = -3.28$	0.021[a]
		$AUC_{0–12h}$ (ng·h/mL)	53.4 (67.6)	86.5 (23.1)	$t_{5.33} = -1.62$	0.16[a]
Dihydroartemisinin	Days 1–3	$AUC_{0–t}$ (ng·h/mL)	732 (11.3)	681 (13.2)	$t_6 = 0.76$	0.48
	Day 1	T_{max} (h)	3.00 (0.98–3.05)	2.44 (1.88–3.00)	$\chi_1^2 = 0.25$	0.62
		C_{max} (ng/mL)	52.2 (25.4)	43.7 (20.0)	$t_6 = 0.89$	0.41
		$AUC_{0–8h}$ (ng·h/mL)	172 (26.6)	138 (12.3)	$t_6 = 1.09$	0.32
	Day 3	T_{max} (h)	3.93 (1.75–4.00)	2.98 (1.92–4.03)	$\chi_1^2 = 0.25$	0.62
		C_{max} (ng/mL)	41.7 (28.5)	66.1 (3.7)	$t_6 = -2.21$	0.069
		$AUC_{0–12h}$ (ng·h/mL)	185 (27.6)	235 (10.6)	$t_6 = -1.17$	0.29
Lumefantrine	Days 1–3	$AUC_{0–t}$ (ng·h/mL)	832,000 (23.4)	712,000 (7.4)	$t_6 = 0.90$	0.40
		$AUC_{0–\infty}$ (ng·h/mL)[b]	828,000 (25.3)	731,000 (6.5)	$t_5 = 0.66$	0.54
		$t_{1/2}$ (h)[b]	196 (24.7)	197 (21.0)	$t_5 = -0.04$	0.97
	Day 1	T_{max} (h)	5.98 (5.00–6.00)	6.01 (6.00–6.02)	$\chi_1^2 = 2.78$	0.096
		C_{max} (ng/mL)	3,510 (99.0)	5,090 (33.8)	$t_6 = -0.59$	0.57
		$AUC_{0–8h}$ (ng·h/mL)	13,100 (100.9)	19,300 (24.0)	$t_6 = -0.63$	0.55
	Day 3	T_{max} (h)	12.00 (3.97–12.20)	8.02 (4.00–12.00)	$\chi_1^2 = 0.44$	0.51

C_{max} (ng/mL)	10,500 (24.5)	7,890 (1.2)	$t_6 = 1.59$	0.16
$AUC_{0–12h}$ (ng·h/mL)	93,800 (37.1)	69,500 (10.6)	$t_6 = 1.11$	0.31

[a] Results from a two-sample t-test assuming unequal variances after Levene's Test for Equal Variances was significant at the 5% level.

Table S4 Post-hoc analysis comparing ruxolitinib pharmacokinetic parameters for the artemether-lumefantrine plus ruxolitinib group (*n* = 6) between day 1 and day 3. Differences over the two time-points were compared non-parametrically using Wilcoxon signed-rank test, and differences of the $\log_{10}$ parameters were compared using a paired t-test and summarized as the ratio of day 3 relative to day 1 and presented as a percent and 95% confidence interval.

Pharmacokinetic parameter	Day 1	Day 3	Relative change (95% CI)[a]	Test statistic	p value
T_{max} (h), median (range)	1.52 (0.98–2.0)	1.98 (1.83–2.0)	0.4% (−0.1, 1.0)	$z = 1.05$	0.29
C_{max} (ng/mL), geometric mean (%CV)[b]	276 (32.7)	126 (24.3)	45.6% (33.7, 61.6)	$t_5 = -6.70$	0.001
$AUC_{0–6h}$ (ng·h/mL), geometric mean (%CV)[b]	839 (20.8)	509 (34.2)	60.6% (46.2, 79.5)	$t_5 = -4.75$	0.005

[a] Change of day 3 pharmacokinetic parameter estimates compared to day 1, presented as the ratio and 95% CI of day 3 relative to day 1.
[b] Parametric analyses calculated on the $\log_{10}$ transformed values of the parameter.
